# 4-(4-Chloro­phen­yl)-*N*-[(*E*)-4-(dimethyl­amino)­benzyl­idene]-1,3-thia­zol-2-amine

**DOI:** 10.1107/S1600536811028078

**Published:** 2011-07-23

**Authors:** S. Vijaya, Vasu, K. V. Arjuna Gowda, T. Narasimhamurthy, R. S. Rathore

**Affiliations:** a Department of Physics, Government First Grade College, Bidadi, Bangalore 560 067, India; b Organic Chemistry Division, Vivekananda Degree Collage, Bangalore 560 055, India; c Department of Physics, Government First Grade College, Mandya 571 401, India; d Materials Research Center, Indian Institute of Science, Bangalore 560 012, India; e Bioinformatics Infrastructure Facility, School of Life Sciences, University of Hyderabad, Hyderabad 500 046, India

## Abstract

The title compound, C_18_H_16_ClN_3_S, adopts an extended mol­ecular structure. The thia­zole ring is inclined by 9.2 (1) and 15.3 (1)° with respect to the chloro­phenyl and 4-(dimethyl­amino)­phenyl rings, respectively, while the benzene ring planes make an angle of 19.0 (1)°. A weak inter­molecular C—H⋯π contact is observed in the crystal structure.

## Related literature

For related structures, see: Lynch *et al.* (1999 ▶; 2002 ▶). For medicinal applications of thia­zole derivatives, see: Misra *et al.* (2004 ▶).
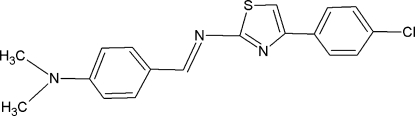


## Experimental

### 

#### Crystal data

C_18_H_16_ClN_3_S
*M* *_r_* = 341.85
Monoclinic, 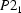

*a* = 6.1169 (7) Å
*b* = 7.4708 (8) Å
*c* = 18.2536 (18) Å
β = 97.975 (11)°
*V* = 826.09 (15) Å^3^
*Z* = 2
Mo *K*α radiation
μ = 0.36 mm^−1^
*T* = 294 K
0.24 × 0.18 × 0.16 mm

#### Data collection

Bruker APEXII CCD diffractometer
Absorption correction: multi-scan (*SADABS*; Bruker, 2004 ▶) *T*_min_ = 0.919, *T*_max_ = 0.945
9063 measured reflections
3242 independent reflections
1355 reflections with *I* > 2σ(*I*)
*R*_int_ = 0.084

#### Refinement

*R*[*F* ^2^ > 2σ(*F* ^2^)] = 0.048
*wR*(*F* ^2^) = 0.062
*S* = 0.78
3242 reflections
210 parameters
1 restraint
H-atom parameters constrained
Δρ_max_ = 0.18 e Å^−3^
Δρ_min_ = −0.20 e Å^−3^
Absolute structure: Flack (1983 ▶), 1483 Friedel pairs
Flack parameter: 0.06 (8)

### 

Data collection: *APEX2* (Bruker, 2010 ▶); cell refinement: *SAINT-Plus* (Bruker, 2010 ▶); data reduction: *SAINT-Plus*; program(s) used to solve structure: *SHELXS97* (Sheldrick, 2008 ▶); program(s) used to refine structure: *SHELXL97* (Sheldrick, 2008 ▶); molecular graphics: *ORTEP-3* (Farrugia, 1997 ▶); software used to prepare material for publication: *SHELXL97* and *PLATON* (Spek, 2009 ▶).

## Supplementary Material

Crystal structure: contains datablock(s) global, I. DOI: 10.1107/S1600536811028078/xu5251sup1.cif

Structure factors: contains datablock(s) I. DOI: 10.1107/S1600536811028078/xu5251Isup2.hkl

Supplementary material file. DOI: 10.1107/S1600536811028078/xu5251Isup3.cml

Additional supplementary materials:  crystallographic information; 3D view; checkCIF report

## Figures and Tables

**Table 1:** Hydrogen-bond geometry (Å, °) *Cg* is the centroid of the C11–C16 ring.

*D*—H⋯*A*	*D*—H	H⋯*A*	*D*⋯*A*	*D*—H⋯*A*
C18—H18*B*⋯*Cg*^i^	0.96	2.73	3.515 (5)	140

Symmetry code: (i) 
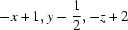
.

**Fig. 1.**
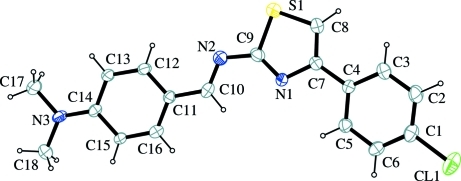
A view of (I) with adopted atom-numbering scheme and non-H atoms shown as probability ellipsoids at 30% levels.

## supplementary crystallographic information

### Comment

The title compound, C_18_H_16_ClN_3_S, (I), is a 2-amino-thiazole derivative. Few structures of such derivatives have been determined (Lynch *et al.*, 1999; 2002) and some of them have been shown to act as inhibitor of cyclin-dependent kinase (Misra *et al.*, 2004). The structure of (I) with adopted atom-numbering scheme is shown in Fig 1.

(I) adopts an extended structure. The thiazole ring is inclined with respect to chlorophenyl and dimethylaminophenyl rings by 9.2 (1)° and 15.3 (1)°, respectively, while both benzene ring planes make an angle of 19.0 (1)°. The dimethylamino group makes an angle of 4.0 (3)° with respect to the adjacent benzene ring. The crystal packing is governed by van der waals interactions only. Short intermolecular C—H···π contact is also observed (Table 1).

### Experimental

A mixture of 2-amino-4-(4-chloro) phenyl thiazole (0.01 mol; CAS No. 2103–99-3) and paradimethyl amino benzaldehyde (0.01 mol) in ethanol (30 ml), and catalytic amount of glacial acetic acid (2 ml) in a clean conical flask was refluxed for 2 h. The resulting mixture was cooled, filtered and dried to get the title compound (m.p. 506–507°C). To obtain the suitable single crystals for X-ray diffraction, (I) was mixed with DMF (30 ml) and heated until completely dissolved. The mixture was left for slow evaporation.

### Refinement

Hydrogen atoms were placed in their stereochemically expected positions and refined with the riding options. Methyl hydrogen atoms were fixed with reference to local electron density map. The distances with hydrogen atoms are as follows: C(aromatic/*sp*^2^)—H = 0.93 Å, *C*(methyl)—H = 0.96 Å, and *U*_iso_ = 1.2 *U*_eq_(parent) [1.5*U*_eq_(parent) for methyl groups].

### Crystal data

**Table:**

C_18_H_16_ClN_3_S	*F*(000) = 356
*M**_r_* = 341.85	*D*_x_ = 1.374 Mg m^−^^3^
Monoclinic, *P*2_1_	Mo *K*α radiation, λ = 0.71073 Å
Hall symbol: P 2yb	Cell parameters from 1055 reflections
*a* = 6.1169 (7) Å	θ = 2.2–29.2°
*b* = 7.4708 (8) Å	µ = 0.36 mm^−^^1^
*c* = 18.2536 (18) Å	*T* = 294 K
β = 97.975 (11)°	Needle, brown
*V* = 826.09 (15) Å^3^	0.24 × 0.18 × 0.16 mm
*Z* = 2	

### Data collection

**Table:**

Bruker APEXII CCD diffractometer	3242 independent reflections
Radiation source: fine-focus sealed tube	1355 reflections with *I* > 2σ(*I*)
graphite	*R*_int_ = 0.084
φ and ω scans	θ_max_ = 26.0°, θ_min_ = 3.0°
Absorption correction: multi-scan (*SADABS*; Bruker, 2004)	*h* = −7→7
*T*_min_ = 0.919, *T*_max_ = 0.945	*k* = −9→9
9063 measured reflections	*l* = −22→22

### Refinement

**Table:**

Refinement on *F*^2^	Secondary atom site location: difference Fourier map
Least-squares matrix: full	Hydrogen site location: inferred from neighbouring sites
*R*[*F*^2^ > 2σ(*F*^2^)] = 0.048	H-atom parameters constrained
*wR*(*F*^2^) = 0.062	*w* = 1/[σ^2^(*F*_o_^2^) + (0.010*P*)^2^] where *P* = (*F*_o_^2^ + 2*F*_c_^2^)/3
*S* = 0.78	(Δ/σ)_max_ = 0.004
3242 reflections	Δρ_max_ = 0.18 e Å^−^^3^
210 parameters	Δρ_min_ = −0.20 e Å^−^^3^
1 restraint	Absolute structure: Flack (1983), 1483 Friedel pairs
Primary atom site location: structure-invariant direct methods	Flack parameter: 0.06 (8)

### Special details

**Table:**

Geometry. All e.s.d.'s (except the e.s.d. in the dihedral angle between two l.s. planes) are estimated using the full covariance matrix. The cell e.s.d.'s are taken into account individually in the estimation of e.s.d.'s in distances, angles and torsion angles; correlations between e.s.d.'s in cell parameters are only used when they are defined by crystal symmetry. An approximate (isotropic) treatment of cell e.s.d.'s is used for estimating e.s.d.'s involving l.s. planes.Weighted least-squares planes through the starred atoms (Nardelli, Musatti, Domiano & Andreetti Ric.Sci.(1965),15(II—A),807). Equation of the plane: m1**X*+m2*Y+m3**Z*=dPlane 1 m1 = -0.44990(0.00160) m2 = -0.87347(0.00086) m3 = -0.18611(0.00175) D = -3.47756(0.01551) Atom d s d/s (d/s)**2 C1 * -0.0082 0.0048 - 1.706 2.911 C2 * 0.0089 0.0046 1.925 3.707 C3 * -0.0021 0.0045 - 0.472 0.223 C4 * -0.0040 0.0039 - 1.036 1.072 C5 * 0.0051 0.0039 1.304 1.700 C6 * -0.0004 0.0040 - 0.096 0.009 Cl1 - 0.0062 0.0013 - 4.835 23.378 C7 - 0.0409 0.0039 - 10.501 110.265 ============ Sum((d/s)**2) for starred atoms 9.623 Chi-squared at 95% for 3 degrees of freedom: 7.81 The group of atoms deviates significantly from planarityPlane 2 m1 = -0.34887(0.00131) m2 = -0.93392(0.00058) m3 = -0.07797(0.00194) D = -2.96690(0.02047) Atom d s d/s (d/s)**2 N1 * 0.0036 0.0034 1.053 1.108 S1 * 0.0002 0.0012 0.201 0.041 C7 * -0.0021 0.0039 - 0.541 0.293 C8 * -0.0007 0.0043 - 0.157 0.025 C9 * -0.0054 0.0042 - 1.283 1.647 N2 - 0.0838 0.0039 - 21.454 460.260 C4 - 0.0329 0.0039 - 8.470 71.739 ============ Sum((d/s)**2) for starred atoms 3.113 Chi-squared at 95% for 2 degrees of freedom: 5.99 The group of atoms does not deviate significantly from planarityPlane 3 m1 = 0.47925(0.00159) m2 = 0.86590(0.00089) m3 = -0.14330(0.00169) D = -0.77606(0.02916) Atom d s d/s (d/s)**2 C11 * 0.0080 0.0041 1.980 3.920 C12 * -0.0093 0.0041 - 2.291 5.251 C13 * 0.0037 0.0043 0.856 0.733 C14 * 0.0043 0.0042 1.010 1.021 C15 * -0.0044 0.0038 - 1.160 1.346 C16 * -0.0009 0.0040 - 0.219 0.048 N3 0.0139 0.0033 4.236 17.942 C10 0.0759 0.0041 18.730 350.815 ============ Sum((d/s)**2) for starred atoms 12.318 Chi-squared at 95% for 3 degrees of freedom: 7.81 The group of atoms deviates significantly from planarityPlane 4 m1 = 0.46397(0.00261) m2 = 0.88241(0.00119) m3 = -0.07795(0.00540) D = 0.57261(0.10767) Atom d s d/s (d/s)**2 N3 * 0.0000 0.0033 0.000 0.000 C17 * 0.0000 0.0040 0.000 0.000 C18 * 0.0000 0.0041 0.000 0.000 C14 - 0.0851 0.0042 - 20.162 406.523 ============ Sum((d/s)**2) for starred atoms 0.000 Dihedral angles formed by LSQ-planes Plane - plane angle (s.u.) angle (s.u.) 1 2 9.17 (0.13) 170.83 (0.13) 1 3 19.04 (0.14) 160.96 (0.14) 1 4 15.20 (0.31) 164.80 (0.31) 2 3 15.27 (0.13) 164.73 (0.13) 2 4 11.51 (0.27) 168.49 (0.27) 3 4 3.96 (0.31) 176.04 (0.31)
Refinement. Refinement of *F*^2^ against ALL reflections. The weighted *R*-factor *wR* and goodness of fit *S* are based on *F*^2^, conventional *R*-factors *R* are based on *F*, with *F* set to zero for negative *F*^2^. The threshold expression of *F*^2^ > σ(*F*^2^) is used only for calculating *R*-factors(gt) *etc*. and is not relevant to the choice of reflections for refinement. *R*-factors based on *F*^2^ are statistically about twice as large as those based on *F*, and *R*- factors based on ALL data will be even larger.

### Fractional atomic coordinates and isotropic or equivalent isotropic displacement parameters (Å^2^)

**Table:**

	*x*	*y*	*z*	*U*_iso_*/*U*_eq_	
S1	−0.58295 (18)	0.55168 (16)	0.68740 (6)	0.0603 (4)	
Cl1	−0.0164 (2)	0.44097 (17)	0.29270 (6)	0.0840 (5)	
N1	−0.2184 (5)	0.4428 (5)	0.64682 (18)	0.0417 (9)	
N2	−0.2363 (6)	0.4507 (5)	0.77983 (19)	0.0541 (10)	
N3	0.3895 (6)	0.3460 (4)	1.0889 (2)	0.0448 (10)	
C1	−0.1183 (8)	0.4555 (6)	0.3769 (2)	0.0504 (13)	
C2	−0.3184 (7)	0.5363 (6)	0.3800 (2)	0.0577 (13)	
H2	−0.4002	0.5824	0.3374	0.069*	
C3	−0.3970 (6)	0.5480 (6)	0.4478 (2)	0.0490 (12)	
H3	−0.5311	0.6045	0.4506	0.059*	
C4	−0.2781 (6)	0.4765 (5)	0.5114 (2)	0.0340 (11)	
C5	−0.0787 (6)	0.3928 (5)	0.5059 (2)	0.0445 (13)	
H5	0.0023	0.3429	0.5478	0.053*	
C6	0.0014 (7)	0.3830 (5)	0.4382 (2)	0.0473 (13)	
H6	0.1356	0.3273	0.4349	0.057*	
C7	−0.3576 (7)	0.4909 (5)	0.5840 (2)	0.0373 (12)	
C8	−0.5606 (6)	0.5520 (6)	0.5945 (2)	0.0512 (12)	
H8	−0.6706	0.5884	0.5571	0.061*	
C9	−0.3158 (6)	0.4695 (6)	0.7050 (3)	0.0491 (13)	
C10	−0.0454 (7)	0.3803 (5)	0.7958 (2)	0.0442 (13)	
H10	0.0264	0.3374	0.7577	0.053*	
C11	0.0633 (7)	0.3650 (5)	0.8714 (3)	0.0369 (11)	
C12	−0.0298 (6)	0.4390 (6)	0.9301 (2)	0.0422 (11)	
H12	−0.1679	0.4928	0.9206	0.051*	
C13	0.0767 (6)	0.4348 (6)	1.0015 (2)	0.0422 (12)	
H13	0.0112	0.4879	1.0391	0.051*	
C14	0.2839 (7)	0.3510 (6)	1.0185 (2)	0.0376 (12)	
C15	0.3763 (7)	0.2732 (5)	0.9598 (2)	0.0415 (12)	
H15	0.5122	0.2158	0.9690	0.050*	
C16	0.2669 (7)	0.2815 (5)	0.8887 (2)	0.0466 (13)	
H16	0.3318	0.2293	0.8508	0.056*	
C17	0.2862 (6)	0.4146 (6)	1.1505 (2)	0.0654 (15)	
H17C	0.1580	0.3443	1.1559	0.098*	
H17B	0.2435	0.5370	1.1411	0.098*	
H17A	0.3891	0.4078	1.1952	0.098*	
C18	0.6027 (7)	0.2620 (6)	1.1087 (2)	0.0608 (14)	
H18A	0.7059	0.3112	1.0790	0.091*	
H18B	0.5894	0.1354	1.1002	0.091*	
H18C	0.6543	0.2836	1.1600	0.091*	

### Atomic displacement parameters (Å^2^)

**Table:**

	*U*^11^	*U*^22^	*U*^33^	*U*^12^	*U*^13^	*U*^23^
S1	0.0511 (8)	0.0798 (10)	0.0498 (9)	0.0126 (8)	0.0057 (7)	−0.0004 (8)
Cl1	0.1220 (12)	0.0840 (10)	0.0528 (9)	−0.0034 (9)	0.0367 (9)	0.0026 (8)
N1	0.045 (2)	0.048 (2)	0.032 (2)	0.010 (2)	0.0036 (19)	−0.003 (2)
N2	0.047 (2)	0.072 (3)	0.044 (3)	0.005 (2)	0.009 (2)	0.002 (2)
N3	0.050 (3)	0.047 (3)	0.041 (3)	0.014 (2)	0.018 (2)	0.001 (2)
C1	0.072 (4)	0.039 (3)	0.043 (3)	−0.006 (3)	0.016 (3)	0.003 (3)
C2	0.074 (4)	0.056 (3)	0.043 (3)	0.009 (3)	0.006 (3)	0.019 (3)
C3	0.052 (3)	0.048 (3)	0.045 (3)	−0.003 (3)	−0.001 (3)	0.007 (3)
C4	0.040 (3)	0.028 (3)	0.035 (3)	−0.005 (2)	0.006 (2)	−0.002 (2)
C5	0.050 (3)	0.044 (3)	0.039 (3)	0.005 (2)	0.005 (3)	0.002 (2)
C6	0.053 (3)	0.041 (3)	0.049 (3)	0.001 (3)	0.015 (3)	−0.007 (3)
C7	0.043 (3)	0.026 (3)	0.042 (3)	−0.002 (2)	0.000 (2)	−0.004 (2)
C8	0.051 (3)	0.059 (3)	0.042 (3)	0.003 (3)	−0.002 (2)	−0.001 (3)
C9	0.048 (3)	0.045 (3)	0.052 (3)	0.009 (2)	−0.001 (3)	0.001 (3)
C10	0.057 (3)	0.040 (3)	0.039 (3)	−0.008 (3)	0.015 (3)	−0.006 (2)
C11	0.039 (3)	0.038 (3)	0.035 (3)	−0.003 (2)	0.008 (3)	−0.001 (2)
C12	0.037 (3)	0.046 (3)	0.046 (3)	0.003 (2)	0.013 (3)	−0.006 (3)
C13	0.041 (3)	0.053 (3)	0.034 (3)	0.006 (3)	0.011 (2)	0.001 (3)
C14	0.046 (3)	0.037 (3)	0.033 (3)	−0.001 (2)	0.016 (3)	0.006 (2)
C15	0.042 (3)	0.045 (3)	0.038 (3)	0.012 (2)	0.006 (3)	−0.008 (3)
C16	0.049 (3)	0.046 (3)	0.047 (3)	0.004 (2)	0.015 (3)	−0.004 (3)
C17	0.066 (3)	0.089 (4)	0.042 (3)	0.018 (3)	0.013 (3)	−0.003 (3)
C18	0.055 (4)	0.074 (4)	0.050 (4)	0.015 (3)	−0.005 (3)	0.001 (3)

### Geometric parameters (Å, °)

**Table:**

S1—C8	1.720 (4)	C7—C8	1.362 (4)
S1—C9	1.733 (4)	C8—H8	0.9300
Cl1—C1	1.741 (4)	C10—C11	1.451 (5)
N1—C9	1.303 (4)	C10—H10	0.9300
N1—C7	1.378 (4)	C11—C16	1.390 (5)
N2—C10	1.277 (4)	C11—C12	1.394 (4)
N2—C9	1.393 (4)	C12—C13	1.375 (4)
N3—C14	1.357 (5)	C12—H12	0.9300
N3—C18	1.447 (5)	C13—C14	1.409 (5)
N3—C17	1.458 (4)	C13—H13	0.9300
C1—C6	1.361 (5)	C14—C15	1.404 (5)
C1—C2	1.373 (5)	C15—C16	1.377 (5)
C2—C3	1.392 (4)	C15—H15	0.9300
C2—H2	0.9300	C16—H16	0.9300
C3—C4	1.388 (5)	C17—H17C	0.9600
C3—H3	0.9300	C17—H17B	0.9600
C4—C5	1.386 (5)	C17—H17A	0.9600
C4—C7	1.477 (4)	C18—H18A	0.9600
C5—C6	1.394 (4)	C18—H18B	0.9600
C5—H5	0.9300	C18—H18C	0.9600
C6—H6	0.9300		
			
C8—S1—C9	88.9 (2)	N2—C10—C11	122.3 (4)
C9—N1—C7	109.8 (3)	N2—C10—H10	118.9
C10—N2—C9	116.7 (4)	C11—C10—H10	118.9
C14—N3—C18	122.9 (3)	C16—C11—C12	116.9 (4)
C14—N3—C17	121.4 (3)	C16—C11—C10	121.9 (4)
C18—N3—C17	115.6 (4)	C12—C11—C10	121.1 (4)
C6—C1—C2	121.5 (4)	C13—C12—C11	121.9 (4)
C6—C1—Cl1	118.9 (4)	C13—C12—H12	119.0
C2—C1—Cl1	119.6 (4)	C11—C12—H12	119.0
C1—C2—C3	118.9 (4)	C12—C13—C14	120.8 (4)
C1—C2—H2	120.6	C12—C13—H13	119.6
C3—C2—H2	120.6	C14—C13—H13	119.6
C4—C3—C2	120.9 (4)	N3—C14—C15	121.5 (4)
C4—C3—H3	119.5	N3—C14—C13	121.0 (4)
C2—C3—H3	119.5	C15—C14—C13	117.5 (4)
C5—C4—C3	118.6 (3)	C16—C15—C14	120.4 (4)
C5—C4—C7	120.0 (4)	C16—C15—H15	119.8
C3—C4—C7	121.5 (4)	C14—C15—H15	119.8
C4—C5—C6	120.6 (4)	C15—C16—C11	122.5 (4)
C4—C5—H5	119.7	C15—C16—H16	118.8
C6—C5—H5	119.7	C11—C16—H16	118.8
C1—C6—C5	119.5 (4)	N3—C17—H17C	109.5
C1—C6—H6	120.3	N3—C17—H17B	109.5
C5—C6—H6	120.3	H17C—C17—H17B	109.5
C8—C7—N1	116.1 (4)	N3—C17—H17A	109.5
C8—C7—C4	124.8 (4)	H17C—C17—H17A	109.5
N1—C7—C4	119.1 (4)	H17B—C17—H17A	109.5
C7—C8—S1	109.7 (3)	N3—C18—H18A	109.5
C7—C8—H8	125.1	N3—C18—H18B	109.5
S1—C8—H8	125.1	H18A—C18—H18B	109.5
N1—C9—N2	130.3 (4)	N3—C18—H18C	109.5
N1—C9—S1	115.5 (3)	H18A—C18—H18C	109.5
N2—C9—S1	114.1 (3)	H18B—C18—H18C	109.5
			
C6—C1—C2—C3	1.7 (7)	C10—N2—C9—N1	−7.6 (7)
Cl1—C1—C2—C3	−179.6 (3)	C10—N2—C9—S1	175.8 (3)
C1—C2—C3—C4	−1.2 (7)	C8—S1—C9—N1	−0.6 (4)
C2—C3—C4—C5	0.0 (6)	C8—S1—C9—N2	176.5 (3)
C2—C3—C4—C7	178.9 (4)	C9—N2—C10—C11	175.4 (4)
C3—C4—C5—C6	0.8 (6)	N2—C10—C11—C16	177.2 (4)
C7—C4—C5—C6	−178.1 (4)	N2—C10—C11—C12	−4.8 (6)
C2—C1—C6—C5	−1.0 (7)	C16—C11—C12—C13	1.9 (6)
Cl1—C1—C6—C5	−179.7 (3)	C10—C11—C12—C13	−176.2 (4)
C4—C5—C6—C1	−0.3 (6)	C11—C12—C13—C14	−1.5 (7)
C9—N1—C7—C8	−0.7 (5)	C18—N3—C14—C15	0.2 (6)
C9—N1—C7—C4	178.3 (4)	C17—N3—C14—C15	−175.3 (4)
C5—C4—C7—C8	−172.4 (4)	C18—N3—C14—C13	−179.8 (4)
C3—C4—C7—C8	8.8 (6)	C17—N3—C14—C13	4.8 (6)
C5—C4—C7—N1	8.8 (5)	C12—C13—C14—N3	−179.8 (4)
C3—C4—C7—N1	−170.0 (4)	C12—C13—C14—C15	0.2 (6)
N1—C7—C8—S1	0.3 (5)	N3—C14—C15—C16	−179.4 (4)
C4—C7—C8—S1	−178.6 (3)	C13—C14—C15—C16	0.6 (6)
C9—S1—C8—C7	0.1 (3)	C14—C15—C16—C11	−0.2 (6)
C7—N1—C9—N2	−175.7 (4)	C12—C11—C16—C15	−1.0 (6)
C7—N1—C9—S1	0.8 (5)	C10—C11—C16—C15	177.0 (4)

### Hydrogen-bond geometry (Å, °)

**Table:**

Cg is the centroid of the C11–C16 ring.

**Table:**

*D*—H···*A*	*D*—H	H···*A*	*D*···*A*	*D*—H···*A*
C18—H18B···Cg^i^	0.96	2.73	3.515 (5)	140

Symmetry codes: (i) −*x*+1, *y*−1/2, −*z*+2.
